# PGT-M, a Useful Tool to Manage the Lynch Syndrome Transmission

**DOI:** 10.3390/ijms242216114

**Published:** 2023-11-09

**Authors:** Ilaria Listorti, Roberta Manzo, Cristiana Arrivi, Cecilia Mencacci, Anil Biricik, Ermanno Greco, Pierfrancesco Greco

**Affiliations:** 1 Center for Reproductive Medicine, Villa Mafalda, 00199 Rome, Italycmencacci@hotmail.com (C.M.);; 2 Faculty of Biosciences and Agro-Food and Environmental Technologies, University of Teramo, 64100 Teramo, Italy; 3 Eurofins GENOMA Group, Molecular Genetics Laboratories, 00138 Rome, Italy; anilbiricik@eurofins.com; 4 Department of Obstetrician and Genecology, Saint Camillus International University of Health and Medical Sciences (Unicamillus), 00131 Rome, Italy

**Keywords:** hereditary cancer syndrome, lynch syndrome, PGT-M, next-generation sequencing, in vitro fertilization

## Abstract

Lynch syndrome is one of the most common hereditary cancer sensitivity syndromes and is caused by autosomal-dominant germline mutations in DNA mismatch repair genes. In patients affected by this syndrome, pre-implantation genetic testing for monogenic disorders (PGT-M) could be the elective technique used to prevent the transmission of this hereditary syndrome to offspring. Notably, despite the severity of the condition, some authors have observed a markedly lower demand for PGT-M in these patients compared to those with other hereditary conditions. A 34-year-old woman with a medical history of Lynch syndrome associated with endometrial cancer came to the Villa Mafalda fertility center in Rome in order to conceive a healthy baby. In a pre-implantation genetic testing for aneuploidy (PGT-A) + PGT-M cycle, eight blastocysts were formed. Six out of eight blastocysts were affected by the same mother syndrome. One of the other two was aneuploid and the other one was a mosaic embryo, which resulted in a healthy pregnancy. The aim of this report is to emphasize the importance of a multidisciplinary approach to managing patients with this condition. In vitro fertilization (IVF), specifically PGT-M, is a tool that allow patients to conceive biological children with lower risk of inheriting the disease.

## 1. Introduction

Hereditary cancer syndromes (HCS) are a diverse group of genetic conditions that are associated with a higher risk of developing cancer during one’s lifespan. Most of these syndromes are distinguished by an autosomal dominant mutation and they usually start early in life due to the presence of pathogenic variants in one or multiple genes inherited from one or more family members. The most common types of hereditary cancer syndromes inherited by women are breast cancer, ovarian cancer and hereditary non-polyposis colorectal cancer (HNPCC), also named Lynch syndrome (LS). People affected by LS have a higher probability of developing colorectal cancer (50–70%), endometrial carcinomas (40–60%), and several other malignancies including cancer of the ovary, stomach, and pancreas [1,2]. LS is caused by autosomal-dominant germline mutations in DNA mismatch repair genes (*MMR* genes). There are five genes involved in the onset of LS: *MLH1, MSH2, MSH6, PMS2, and EPCAM*. LS carriers inherit variants of one of these genes or carry inherited epigenetically silenced variants of them [3]. The rise of further somatic mutations will cause the spread of malignancies, also causing genomic instability.

The most common germline mutations are present on *MLH1* and *MSH2* genes, and the less frequent are on the *MSH6* and *PMS2* genes [4,5,6,7,8,9]. Mutations in these genes impair the function of MMR proteins, which normally recognize and repair mismatched nucleotides and insertion/deletion loops caused by slippage of DNA polymerase [10,11].

The assessment of hereditary cancer risk is crucial in recognizing people who have a higher risk of contracting a specific type of cancer and to educating them about the cause–effect relationship that the potential disease entails. The patient evaluation—performed by a multidisciplinary team of gynecologists and geneticists—consists of collecting data and information about the patient and his/her family members, including their pathology, and in taking into consideration other cancer-related risk factors. If the assessment conducted shows a potential susceptibility to a specific type of hereditary cancer syndrome, then that evaluation needs to be further conducted by referring the patient to an oncologist, to a specialist in cancer genetics, and/or to a specialist in reproductive medicine. This further study will lead to genetic cancer screening, showing which measures are right for reducing the potential risks related to cancer.

To reduce the risk of contracting gynecological cancer, female carriers (starting at 30–35 years) should undergo comprehensive screening comprehensive via transvaginal ultrasound and endometrial biopsy, with potential treatments up to a total hysterectomy and bilateral salpingo-oophorectomy [12]. It is remarkable that, although this syndrome is very severe, it still remains poorly diagnosed [13].

In individuals affected by HCS, parenting or fertility preservation are characterized by specific implications: firstly, the potential transmission of the mutation to the offspring must be considered, and, secondly, the likelihood of fatal recurrences upon the growth of the child must be assessed. In these circumstances, pre-implantation genetic testing for monogenic disorders (PGT-M) could be the elective technique for application to clinically severe diseases with a high penetrance to prevent the transmission of this hereditary syndrome to offspring.

This study aims to report the case of a woman affected by LS, who underwent an in vitro fertilization (IVF) cycle associated with PGT-M, to emphasize the importance in these patients of receiving a multidisciplinary consultation. Such an occurrence also includes experts in genetics and in reproductive medicine, especially for people in reproductive age. As a matter of fact, a very crucial role is played by genetic counseling prior to and after the genetic testing in order to talk to the patients to better clarify the thinking behind any types of genetic testing, reveal the tests outcomes, and explore the possibility of the types of the cancer-related risks. Most of all, genetic counseling is crucial to educating the patients about the syndrome and about what will come next.

## 2. Case Presentation

A 34-year-old woman with a medical history of Lynch syndrome associated with endometrial cancer came to the Villa Mafalda IVF clinic in Rome in order to conceive a healthy baby.

At the time of the patient’s Lynch syndrome diagnosis, it was revealed through personal and family medical histories of her family there was a nucleotidic substitution a>t in the 5 intron of the *MSH2* gene in the position c.942+3 (called: c.942+3 a>t). This germinal mutation, already described in the literature as pathogenic, caused the “skipping in-frame” of the whole exon 5 on the mRNA. The presence of this mutation determines a predisposition to colorectal and extra-colon cancer, typical of HNPCC syndrome.

To validate and confirm the presence of this mutation, the patient went through several medical tests. First, we ran a molecular exam on the blood sample to search for the mutation on the corresponding fragment of the exon 5 of the MSH2 gene (and of the nearby intronic regions). Applying the SSCP (single-strand conformation polymorphism) technique to the fragment of the *MSH2* gene, which includes the 5 exon and its intronic regions, it was found that the patient had the variant present in her family. Furthermore, we also performed the amplification of the DNA sequencing and it was found that the patient showed the nucleotidic substitution a>t in the intron 5 of the MSH2 gene in position c.942+3 (mutation called c.942+3 a>t). So, the results obtained from these techniques showed that the patient had the germinal mutation c. 942+3 a>t on the MSH2 gene. This was the reason why the patient was predisposed to show the phenotype typical of the hereditary non-polyposis colorectal cancer—HNPCC syndrome. 

The patient presented a clear cell endometrial carcinoma treated with 160 mg (Megace^®^) of megestrol acetate. Clear cell carcinoma of the endometrium is a very aggressive and uncommon carcinoma histotype, accounting for 1–5.5% of all endometrial carcinomas [14]. An endometrial carcinoma appears as a combination of papillary (small round papillae lacking overt stratification), tubulocystic and/or solid architectural patterns, with cuboidal or polygonal cells containing nuclei with a variable degree of pleomorphism. Ovarian and endometrial clear cell carcinomas have been shown to have highly similar gene expression, as well as proteomic, morphologic, and immunohistochemical profiles. The typical immunohistochemical profile of clear cell carcinoma is HNF1B-positive, napsin A-positive, ER-negative, PR-negative and p53-wild-type. HNF1B has high sensitivity for endometrial clear cell carcinoma, but its specificity is lower than that in ovarian clear cell carcinoma. Aberrant mutation-type p53 immunohistochemical expression is seen in up to one third of otherwise typical clear cell carcinomas, and these cases are morphologically indistinguishable from p53-wild-type cases [14].

The patient and her husband, who was tested and did not carry the Lynch syndrome mutation, decided to conceive a healthy baby via in vitro fertilization (IVF) with a PGT-M + PGT-A cycle. They decided this in order to prevent her future child from dealing with the same type of syndrome that affects her from. Controlled ovarian stimulation (COS) was performed using an individualized GnRh antagonist protocol [15,16,17]. Recombinant gonadotrophins were administered according to baseline characteristics from the 2nd day of the menstrual cycle. Ovarian response was monitored by checking LH and E2 levels in the serum and this was performed via an ultrasound measurement of follicular size every 2 or 3 days. The doses were adjusted according to the patient’s response. We administered 0.3 mg of GnRH agonist to achieve final oocyte maturation and, 35–36 h later, oocyte retrieval was performed. She obtained 25 oocytes, and 12 were in the second metaphase. Denudation and intracytoplasmic sperm injection (ICSI) of matured oocytes were performed according to standard laboratory procedures, as described elsewhere [18]. After ICSI, embryos were incubated in sequential media (G-1™ plus, G-2™ plus Vitrolife, Göteborg, Sweden) in an EmbryoScope+ time-lapse system (Vitrolife), at 37 °C, 5% O_2_, and 6% CO_2_ until the blastocyst stage (day 5/day 6). On day one, we performed a check of fertilization (eleven oocytes resulted in fertilization). On day three, a media changeover was performed according to standard laboratory procedures. The blastocysts were assessed according to Gardner and Schoolcraft (1999) [19]. They were classified for the inner cell mass (ICM) and trophectoderm (TE) quality into excellent- (AA), good- (AB/BA), average- (BB/AC/CA), and poor-quality (CC/BC/CB) groups [20]. Eight blastocysts were formed out of eleven embryos and their qualities were: one was excellent; one was good; three were average; and three were of poor quality. The trophectoderm biopsy was performed in accordance with the laboratory protocol, as reported elsewhere [17]. Vitrification was carried out using the Kuwayama protocol [21] using a cryotop^®^ (Fuji, Japan Kitazato) device. PGT-M + PGT-A was performed in accordance with the laboratory protocol. After the whole-genome amplification (SurePlex DNA Amplification System, Illumina Inc., San Diego, CA, USA) of biopsied trophectoderm cells, we performed direct mutation testing via the minisequencing method and haplotype analysis via multiplex PCR amplification of STR markers. These methods, previously selected during pre-clinical set-up study, were performed as described elsewhere [22,23] in order to reveal if any of the embryos were affected by HNPCC syndrome like their mother. The low-pass next-generation sequencing method of detecting the ploidy status of embryos that became wild-type due to the disease-causing mutation has also been applied according to the manufacturer’s protocol (Veriseq-PGS Illumina Inc., USA). Unfortunately, however, 6 out of 8 blastocysts were affected by the same syndrome. One of the other two was aneuploid (one of the poor-quality blastocyts). In the the other one, however, the only excellent blastocyst produced was a mosaic embryo (monosomy for the 13th chromosome-30%) (Table 1).

**Figure 1:**
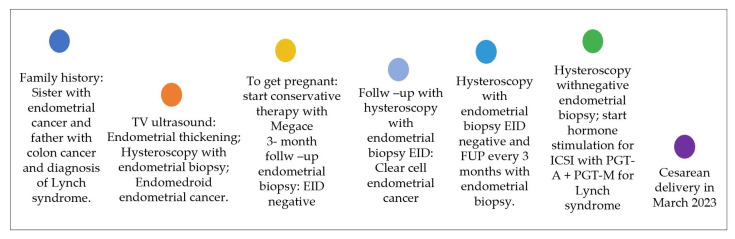
Summary of medical history.

In accordance with the center procedures, the couple went through genetic counseling to decide about the transfer of the mosaic blastocyst. The presence of a mosaic embryo could signify that the embryo was in a chromosomal rescue phase and so, that the fetus could have normal chromosomal assets; or that, if the anomaly presented in the embryo was present also in the fetus, it could not have been compatible with a developmental pregnancy. The couple decided to go through the embryo transfer. Prior to frozen–thawed single-embryo transfer, the patient went through endometrial preparation, combining GnRH agonist and estrogen pills according to the patient’s menstrual cycle. The blastocyst was transferred two hours post-warming in G-2™ plus media (Vitrolife, Göteborg Sweden) and, to maximize the chance of embryo implantation, prior to the transfer, it was incubated in equilibrated hyaluronan-enriched transfer medium for 30 min, as recommended by the manufacturer (EmbryoGlue^®^, Vitrolife, Göteborg Sweden). The use of EmbryoGlue^®^ media favors embryo implantation into the uterus thanks to the presence of hyaluronic acid (HA), which is one of the major macromolecules present in the female reproductive tract. The embryo transfer procedure was carried out with a Wallace catheter (Smiths Medical^®^, Dublin, Ireland) under direct ultrasound guidance, as previously described [24]. To verify if the embryo implantation occurred, the serum β-hCG levels were analyzed. A clinical pregnancy was established by the presence of an ultrasound visualization of the gestational sac with the fetal heartbeat. Due to the presence of a mosaic embryo, the patient underwent amniocentesis at 16 weeks to check the fetal karyotype. The test was normal, and they conceived, via cesarean section-due to the breech position of the baby, a healthy baby boy. The patient medical history is summarized in the figure below (Figure 1).

## 3. Discussion

Approximately 5–10% of cancers occur in the context of HCS. Among HCS, hereditary breast and ovarian cancer syndrome and Lynch syndrome are the two most common, affecting about 1 in 300–500 and 1 in 400–500 individuals, respectively, in the West [2]. The evaluation of hereditary cancer syndrome plays a key role in the recognition of people with higher risk of contracting and developing a specific type of cancer. In this type of evaluation, genetic testing is crucial because it analyzes a series of multiple genes using next-generation sequencing technology (NGS). Thanks to this technique, geneticists are more likely to find specific variants of pathogenic nature that could be associated with a specific cancer syndrome or family cancer phenotype. Furthermore, for people affected by this type of syndromes and who are looking to conceive, preimplantation genetic testing for monogenic/single-gene disorders (PGT-M) today represents a well-established alternative to invasive prenatal diagnosis. This method identifies embryos carrying monogenic disorders or mutations associated with a higher risk of developing pathological conditions, such as hereditary cancer syndromes (HCS) during the lifespan. Embryos obtained through an IVF cycle are biopsied and genetically tested to allow for the transfer of only those which are not affected, or at least to favor the transfer of those not affected in the first place.

Notably, despite the severe condition associated with the LS, Dallagiovanna et al. (2022) [25] observed a markedly lower demand for PGT-M in these patients compared to those with other hereditary syndromes. The European Society for Human Reproduction and Embryology (ESHRE) PGT-M consortium for the years 2016–2017 [26] and for the year 2018 [27] confirm these data without reporting LS as one of the top ten PGT-M indications.

Of note, the acceptance of PGT-M may not strictly depend on the severity of the disease, but also on the patient’s personal history, as well as religious and ethical beliefs. One major ethical concern with this technology is what happens to the embryos that display mutations. While most people choose not to transfer genetically abnormal embryos, those following their religion or ethical belief would often instead prefer to go ahead with the embryo transfer because their religious belief dictate equal respect for embryos [28,29]. Therefore, considering these multidisciplinary issues, health care professionals not only should guide their patients with HCS toward oncological strategies, but should also inform them on the reproductive options available concerning the PGT technology and provide complete and accurate information, in accordance with the national legislation, about the legal and ethical responsibilities towards the developed embryos.

Furthermore, in occurrences such as the one reported in this case report, where the only viable embryo was mosaic, the role of the geneticist is crucial. Greco et al. (2015) [30] reported for the first time the birth of a healthy baby after a mosaic embryo transfer. Embryo mosaicism is a phenomenon characterized by the presence of two genetically different cell lineages, typically one with a chromosome abnormality and another one with normal chromosomal assets [31,32,33]. Arguments remain on the developmental potential of mosaic embryos: the presence of a mosaic embryo could signify that the embryo was in a chromosomal rescue phase and so that the fetus could have normal chromosomal assets [34,35,36]. On the other hand, several case reports exist confirming the presence of mosaicism in babies born following mosaic embryo transfer [37,38]. Consequently, many doubts are still existent about the embryo’s developmental potential and its competence to guarantee a healthy pregnancy according to the percentage and the type of mosaicism.

## 4. Conclusions

Detecting Lynch syndrome is often hard due to the absence of a complete family history information and because there may not be phenotypical evidence typical of the syndrome, such as diffuse polyposis. Nevertheless, early diagnosis is crucial to recognizing patients at high risk of developing the syndrome and which ones will require intensive cancer surveillance. Colonoscopic screening and polypectomy are essential to increasing the chance of the patient’s survival and to reducing the incidence of colorectal tumors [39]. Moreover, in young women, in order to reduce the risk of contracting gynecological cancer, it is advised to undergo a comprehensive screening of a transvaginal ultrasound and endometrial biopsy until total hysterectomy and bilateral salpingo-oophorectomy [12].

In conclusion, Lynch syndrome is a genetic cancer syndrome that requires multidisciplinary education by physicians: oncologists should work closely with the reproductive specialists and geneticists to give the full picture of the patient’s case. Although, as reported in ESHRE-PGT consortium for the years 2016–2018, there is a low demand for the use of PGT-M in LS and IVF patients IVF is a very efficient tool that allow patients to conceive biological children with lower risk of inheriting the disease, exactly like the case mentioned in this case report. However, health care providers need, also, to consider the individual religious and ethical beliefs of the patient and to inform them about the implications of this technology when appropriate.

## Figures and Tables

**Table 1:** Summary of blastocysts classification and PGT-M + PGT-A diagnosis.

Blastocyst No.	Grade	Final Diagnosis
1	4AA	Not Affected + Mosaicism (monosomy for the 13th chromosome, 30%)
3	3CC	Not Affected + Aneuploidy
5	3BB	Affected
6	4BB	Affected
7	4AB	Affected
8	3BB	Affected
9	4CC	Affected
10	3CC	Affected

## Data Availability

The data presented in this study are available on request from the corresponding author. The data are not publicly available due to privacy.
